# The effects of traditional Chinese medicine and dietary compounds on digestive cancer immunotherapy and gut microbiota modulation: A review

**DOI:** 10.3389/fimmu.2023.1087755

**Published:** 2023-02-06

**Authors:** Xiaoli Feng, Zhenhao Li, Weihong Guo, Yanfeng Hu

**Affiliations:** ^1^ Stomatological Hospital, Southern Medical University, Guangzhou, China; ^2^ Department of General Surgery, Nanfang Hospital, The First School of Clinical Medicine, Southern Medical University, Guangzhou, China

**Keywords:** digestive cancer, immunotherapy, traditional Chinese medicine, dietary compounds, microbiota, inflammatory factor, SCFAs

## Abstract

Digestive tract-related cancers account for four of the top ten high-risk cancers worldwide. In recent years, cancer immunotherapy, which exploits the innate immune system to attack tumors, has led to a paradigm shifts in cancer treatment. Gut microbiota modification has been widely used to regulate cancer immunotherapy. Dietary compounds and traditional Chinese medicine (TCM) can alter the gut microbiota and its influence on toxic metabolite production, such as the effect of iprindole on lipopolysaccharide (LPS), and involvement in various metabolic pathways that are closely associated with immune reactions. Therefore, it is an effective strategy to explore new immunotherapies for gastrointestinal cancer to clarify the immunoregulatory effects of different dietary compounds/TCMs on intestinal microbiota. In this review, we have summarized recent progress regarding the effects of dietary compounds/TCMs on gut microbiota and their metabolites, as well as the relationship between digestive cancer immunotherapy and gut microbiota. We hope that this review will act as reference, providing a theoretical basis for the clinical immunotherapy of digestive cancer *via* gut microbiota modulation.

## Introduction

Gastrointestinal cancers, including colorectal and gastric cancer, are among the top five cancer types with the highest mortality rates according to data published by the World Health Organization in 2021 (1). Although significant progress has been made in cancer treatment, improving cancer survival and life expectancy remains a challenge worldwide (2). In the past decade, immune checkpoint blockade (ICB) therapy, which interferes with the interaction between immune checkpoints and receptors, has demonstrated promising therapeutic effects in cancer treatments (3). Antibodies targeting programmed cell death 1 (PD-1), programmed cell death ligand 1 (PD-L1), and cytotoxic T lymphocyte-associated antigen-4 (CTLA-4) have achieved early success in clinical trials, inducing durable remission in various tumor types (4–6). Unfortunately, effective immunotherapy is limited in most patients owing to the immunosuppressive tumor environment, benefitting only 10–40% of patients. In particular, driver gene mutations (7), low tumor-infiltrating lymphocytes (TIL) (8), defects in the antigen presentation process (9), and T cell function loss and failure inhibit immunotherapy effects. Therefore, the efficacy of immunotherapy in tumor treatment requires further improvement.

Hundreds of trillions of microbes make up the gut microbiome, which is the largest microbial community in the human body. Gut microbiota maintain the physiochemical conditions of the gut and aid host digestion, nutrient metabolism, toxin neutralization, and resistance to parasites (10, 11). *Actinobacteria*, *Bacteroidetes*, *Firmicutes*, and *Fusobacteria* constitute the majority of the human gut microbiota (12). Fungi such as *Aspergillus* and *Candida* are also present in the gut microbiome (13). In addition to genetic factors and maternal status during pregnancy, acquired environmental factors affecting gut microbiota include diet (14, 15), lifestyle choices (16), and emotions (17). Previous studies have demonstrated that gut microbiota perturbations influence digestive cancer immunotherapy development. For example, microbiota can ameliorate immunosuppression by altering the tumor microenvironment (18, 19). *Lactobacillus* and *Bifidobacterium* reduce the polarization of invasive monocytes to M2 macrophages and increase M1 phenotype development by upregulating immune factors such as interleukin (IL)-10, thereby inhibiting immune escape and further reducing digestive tumor growth and metastasis (20). Furthermore, products metabolized by microbiota are also involved in immune regulation. Short-chain fatty acids (SCFAs) restrain specific enzymes involved in the transmission of genetic materials that alter the metabolism and gene regulation of immune cells, contributing to a positive impact on digestive tumor therapy. Moreover, inosine from *B.* longum promotes T helper (TH)1 cell differentiation and enhances the therapeutic effect of ICB, mediated by the T cell-specific adenosine A2A receptor (A2AR) (21).

Dietary compounds such as dietary fiber, flavonoids, alkaloids, and polysaccharides are bioactive metabolites which play a crucial role in maintaining health and adjusting physiological functions (Food and Drug Administration and HSS, 2016). They can transform intestinal microbial components and produce intestinal metabolites, such as hydrogen, methane, SCFAs and B vitamins after microflora fermentation in the large intestine (22). Traditional Chinese medicine (TCM) has been used therapeutically for several millennia. Nearly 100 species of Chinese herbal crude drugs and their preparations are widely used in the medical industry and are included in the *European Pharmacopoeia* and the *United States Pharmacopoeia* (23). The chemical composition of TCMs is complex including sugars, amino acids, proteins, vitamins, and dietary compounds originating from plant cell walls (24). Most TCMs are administered orally and interact with the microflora, which affects immune function and influences cancer immunotherapy (25). Recent studies have shown that dietary compounds/TCMs can modulate intestinal microbiota structure and metabolic pathways, improve the composition of the tumor immune microenvironment, and show potential in turning a cold tumor (i.e. immune desert) into a hot tumor (i.e. immune-infiltrated) to enhance immunotherapy efficacy (26–28). Some dietary compounds/TCMs can be fermented or converted by the gut microbiota to form bioactive components. For example, puerarin and isoflavone glycosides can be metabolized by gut flora into daidzein and pistil isoflavones that are more effective than their precursors (29).

**Figure 1:**
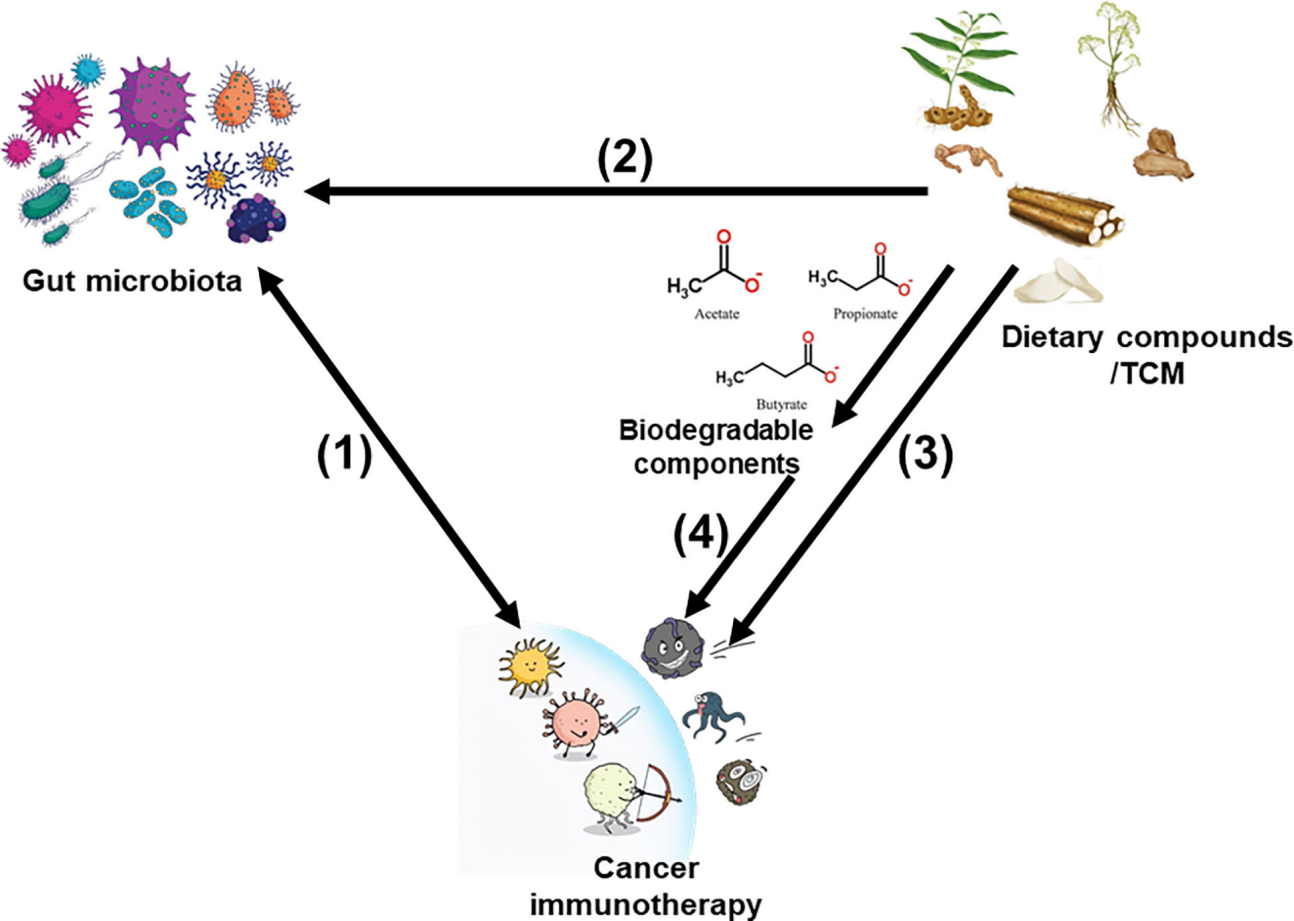
Links betweencancer immunotherapy, dietary compounds/TCM, and gut microbiota. (1) The relationship between gut microbiota and cancer immunotherapy is bidirectional. (2) Dietary compounds/TCM modulate gut microbiota composition and metabolism. (3) Dietary compounds/TCM can directly improve the immune system. (4) Biodegradable components produced by intestinal microbiota derived from dietary compounds/TCM also improve cancer immunotherapy effects.

Although ICB remains one of the most commonly used techniques to clinically treat cancer, several studies have used dietary compounds/TCMs as a supplementary treatment choice to explore new immunotherapies with high efficiency and few adverse reactions, and improve the survivability and quality of life of patients with tumors (30–34). We have summarized these dietary compounds/TCMs currently under preclinical or clinical research and critically evaluated their future development potential to provide new ideas for tumor immunotherapy through the intervention of microflora. **Figure 1** illustrates the links between digestive cancer immunotherapy, dietary compounds/TCMs, and the intestinal microbiota.

## Gut microbiota involved in digestive tract tumorigenesis

Many studies have demonstrated the alteration of gut microbiota in digestive cancer patients. The efforts to identify the specific processes by which the gut microbiota contributes to the development of cancer have received a lot of attention.

### Gastric cancer

Numerous virulence factors are produced by *Helicobacter pylori*, including the oncoproteins cytotoxin-associated gene A (CagA) and vacuolating cytotoxin A (VacA), which have been identified as the primary virulence factors involved in the pathogenesis of gastric cancer (35). Additionally, *Helicobacter pylori* causes gastric atrophy and gastric acid shortage, which promote excessive microbial growth in the stomach, resulting in more nitrogen derivatives in the diet that can be converted into carcinogens (36).

### Colon cancer

It was reported that *Fusobacterium* are abundant in patients with colorectal cancer (CRC) (37). Surface adhesion protein (FadA), the primary factor controlling the adherence and invasion of *Fusobacterium*, can bind to β-catenin and lead to its activation, which in turn triggers inflammation and tumor growth (38). Additionally, many bacterial metabolites may also cause genomic instability, leading to tumorigenesis. For instance, Enterobacteriaceae produce colibactin, which stimulates the overexpansion of intestinal epithelial cells and aids in the development of CRC by generating DNA damage, mutation, and genomic instability (39). *Enterococcus faecalis* can induce double-stranded DNA breaks and promote the development of CRC in mice by producing superoxide free radicals (40). Similarly, secondary bile acids produced by gut bacteria can affect the mitotic process of intestinal epithelial cells, induce DNA damage, and increase the risk of CRC (41). The metabolism of gut bacteria also produces other cancer-promoting substances such as glucuronidase that can transform the precarcinogens in food or drugs into carcinogens (42); or produce carcinogenic chemicals such as N-nitroso compounds (NOCs) (43) and hydrogen sulfide (H_2_S) (44).

### Hepatocellular carcinoma

A growing body of literature points to an increased abundance of lipopolysaccharide (LPS)- producing bacteria (*Neisseria*, *Enterobacteriaceae*, and *Vermicella*) in patients with hepatocellular carcinoma (HCC) (45–47). It is reported that LPS can activate the toll-like receptor (TLR) 4 and NF-κB pathways and trigger the production of cytokines such as TNF-α and IL-6 that promote cancer progression (48). Secondary bile acids can make the intestinal mucosa more permeable, allowing intestinal pathogens to translocate to the liver and cause HCC (49, 50). Through the portal venous system, the liver is connected to intestinal bacterial components and their metabolites, which may cause inflammatory alterations, hepatotoxicity, and finally, HCC. For instance, alterations in the gut microbiota raised the levels of hepatobiliary acid, which caused hepatocarcinogenesis in an obesity-induced human liver cancer xenograft mice model (51).

### Esophageal cancer

*Fusobacteria* is one of the most common microorganisms found in the esophagus (52). Matrix metalloproteinases (MMPs) can be secreted by intestinal epithelial cells when *Fusobacterium* is present (53). Activated MMPs can encourage the growth of tumor cells by increasing the breakdown of the esophageal extracellular matrix, stimulating tumor angiogenesis, and controlling cell adhesion and motility (54). *Fusobacterium* simultaneously triggers the IL-6/p-STAT3/c-MYC signaling pathway and encourages M2-type differentiation of macrophages through a TLR4-dependent mechanism, promoting tumor growth (55). Therefore, *Fusobacterium* is a potential target for the treatment of esophageal cancer.

## Digestive cancer immunotherapy *via* gut microbiota modulation

**Figure 2:**
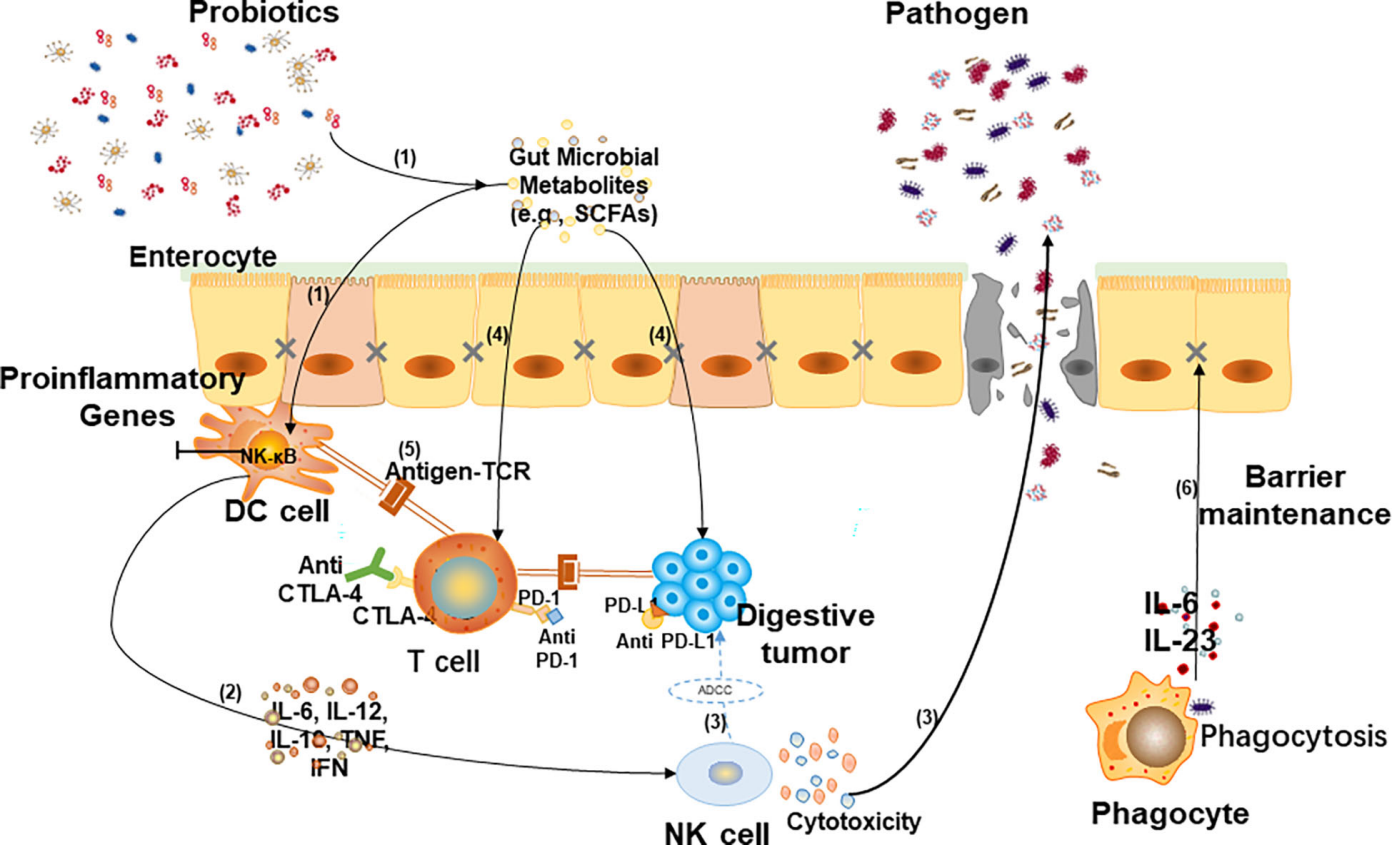
Relationship between gut microbiota and digestive cancer immunotherapy. (1) When tumors arise, gut flora and its metabolites (such as short-chain fatty acids) activate dendritic (DC) cells. (2) Cytokines are involved in activating NK cells and promoting the migration of NK cells to tumor sites. (3) Killer (NK) cells can directly kill tumor cells *via* antibody-dependent cellular cytotoxicity (ADCC) and inhibit pathogenicity through cytotoxicity. (4) Some metabolites shape the immune system by regulating T cell differentiation and also participate in tumor-killing by interacting with host cell surface receptors. (5) Anti-cytotoxic T lymphocyte-associated antigen-4 (CTLA-4) enhance the interaction between DC cells and T cells, and anti-programmed cell death ligand 1 (PD-L1) stimulate T cell immune response to promote immunotherapy. (6) Phagocytes eliminate pathogens by phagocytosis and secret cytokines to recover intestinal barrier dysfunction.

Due to the resistance of a large number of patients to chemotherapy drugs, gut microbiota- mediated immunotherapy has become one of the most promising methods in cancer research in recent years (56). The gut microbiota plays a key role in the occurrence and development of digestive cancer by regulating metabolism, immune response and inflammation (57). The relationship between gut microbiota and digestive cancer immunotherapy is shown in **Figure 2**.

### Gastric cancer

*Bifidobacterium* augmented anti-CTLA-4 checkpoint blockade in mouse models (58), whereas antibiotic-treated or sterile mice showed no response to this blockade. More abundant bacterial species such as *Enterococcus faecium* enhance anti-PD-L1 treatment in mouse models (18). Recent research has shown that the use of probiotics as adjunctive therapy for *Helicobacter pylori* infection can effectively inhibit the progression of gastric cancer (59, 60). Probiotics administered to patients with gastric cancer following total gastrectomy were found to improve immune function and reduce inflammation (61).

### Colon cancer

*Eubacterium*, *Lactobacillus*, and *Streptococcus* can release multiple metabolites that influence the immune system, such as SCFAs, which are positively connected to the anti-PD-1/PD-L1 response (62). SCFAs alter inflammation, including the activation, proliferation, and differentiation of anti-inflammatory T regulatory (Treg) cells or pro-inflammatory Th1 and Th17 cells, and affect the polarization of pro-inflammatory M1 and anti-inflammatory M2 macrophages (63). Furthermore, SCFAs cause tumor growth by activating mitogen-activated protein kinase and PI3K (phosphatidylinositol-3-kinases) signaling by increasing somatomedin C (IGF-1) levels. In addition, virulence factors are important components of gut flora that affect CRC immunotherapy (64). TcdB produced by *Clostridium difficile* inhibits TH and memory B-cell differentiation (65). Therefore, modulating *the Clostridium difficile* count is beneficial for immune recovery. Interestingly, in some microflora such as *Bacteroides fragilis*, the cross-reaction between bacterial antigen and tumor neoantigen can activate antitumor T cells (66). In contrast, the enterotoxins BFT (*Bacteroides fragilis* enterotoxin) and IL-17 produced by *Bacteroides fragilis* induce the differentiation of monocytic myeloid-derived suppressor cells into intestinal epithelial cells, which can selectively upregulate arginase 1 (Arg1) and type 2 NO synthase (NOS2) to produce NO, inhibit T cell proliferation, and promote CRC generation (67).

### Hepatocellular carcinoma

The function of dendritic cells can be enhanced by oral *Bifidobacterium* administration, which increases CD8^+^ T cell accumulation in HCC tissue (68). Similarly, SagA, an enzyme expressed by *Enterococcus faecium*, enhances the effect of immunotherapy in HCC (69). Microbial metabolites, including amino acid derivatives and secondary BAs, are also involved in immunoregulation. *Lactobacillus* is able to convert tryptophan into indole and its derivatives, which are major aromatic hydrocarbon receptors (AHRs) that play a pivotal role in intestinal immunobarrier function (70). In addition, the bacterial metabolites lactic acid and pyruvate, enhance the immune response by inducing GPR31-mediated dendritic cell differentiation (71).

### Esophageal cancer

Immune checkpoint inhibitor (ICI) therapies have been evaluated for their effect on specific microbes in esophageal cancer, including cytotoxic T lymphocyte-associated protein 4 (CTLA-4) and programmed cell death 1 (PD-1)/PD-1 ligand (PD-L1) inhibitors (72). Vetizou et al. found that anti-CTLA-4 therapy was effective only when *B. fragilis* and/or *B. thetaiotaomicron* and *Burkholderiales* populations were present and are therapeutic when T cells are specific for *B. fragilis* and *B. thetaiotamicron* (58). In addition, the reintroduction of *B. fragilis* cells and/or polysaccharides or adoptive transfer of *B. fragilis*-specific T cells restored therapeutic efficacy and reduced immune-mediated colitis through activation of Th1 cells with cross-reactivity to bacterial antigens and tumour neoantigens. These results indicate that reconstruction of intestinal flora is beneficial for esophageal cancer treatment through immune regulation.

## Interactions between dietary compounds and gut microbiota

### Dietary compounds affect microbiota metabolites

#### SCFAs

Several epidemiological studies have shown that in inflammatory disease and digestive cancer, particularly gastric and colon cancer, the incidence rate is related to SCFA shortage in the diet (73). SCFAs, mainly composed of acetate, propionate, and butyrate, inhibit histone deacetylase (HDAC) and G protein-coupled receptor (GPCR) activation pathways to induce phagocytes to secrete chemokines and anti-inflammatory factors, block phagocytes from releasing tumor necrosis factor (TNF), and promote T lymphocyte proliferation and differentiation for the treatment of tumors (74, 75). Notably, when treating intestinal inflammation with dietary fiber, high-dose butyrate caused by continuous inulin intake may cause stagnation of colonic epithelial stem cell proliferation and even inflammation and obstruction of the urinary system, thus damaging the immune system (76). This indicates that the dietary compound dose also affects the metabolic function of microbiota.

#### Tryptophan

After treatment with active ginseng polysaccharides, indoleamine 2,3-dioxygenase (IDO) activity was substantially reduced, causing the microflora to produce more L-tryptophan and less L-kynurenine. Therefore, dietary compounds that lower IDO activity to modulate microfloral metabolites are a potential route of anti-PD-1 therapy resistance (76). Furthermore, intestinal microbes can break down TRP to produce indole-containing metabolites, which modulate the host immune system by activating the ligand-gated transcription factor AHR.

#### Secondary bile acids

The intestinal microbes convert primary bile acids into secondary bile acids (lithocholic and deoxycholic acids) in the large intestine. Two primary bile acid receptors, farnesoid X receptor (FXR) and G-protein coupled bile acid receptor (TGR), modulate the synthesis, metabolism, and redistribution of bile acids through interactions with the gut microbiota (77). The development of *Romboutsia* following treatment with *Tremella fuciformis* polysaccharides can increase the production of deoxycholic acid in the intestine and alter the metabolism of bile acids, which has a considerable influence on the treatment of colitis (78). Kaempferol has been used to increase the expression of sterol 27-hydroxylase (CYP27A1) and FXR to counteract the declining trend of deoxycholic acid (79). This decreased the tumor burden in Apc^Min/+^ mice and repaired the intestinal barrier. Moreover, the increase in secondary bile acids was closely related to the gut microbiota as demonstrated by the greater number of species with anticancer capabilities in the kaempferol therapy group (80).

#### Pyruvate and lactic acid

The gut microorganisms can ferment dietary fiber to produce pyruvate and lactic acid. When the expression of GPR81 and Wnt3 lactic acid-specific receptors in Paneth cells and stromal cells is increased, lactic acid stimulates the proliferation of intestinal epithelial stem cells and prevents intestinal damage. Lactic acid also affects the expression of CX3CR1, a phagocyte in the lamina propria that modulates intestinal immune function (80). Codium fragile extract boosted the percentage of beneficial bacteria and decreased the degree of pyruvate fermentation and glycolysis, reducing the inflammatory reaction induced by a high-fat diet (81). Additionally, to reduce intestinal inflammation and treat metabolic disorders of the microbiota, polysaccharides extracted from *Rosa Roxburghii* Tratt (RTFP) can decrease the Firmicutes/Bacteroides ratio, lower the levels of d-lactic acid and LPS, and suppress the TLR4/NF-κB signaling pathway. It has been reported that RTFP can be administered as a natural anti-inflammatory agent to minimize colitis caused by chronic obesity (82).

### Dietary compounds affect microbiota composition

According to their natural properties, intestinal microbiota can be divided into nine phyla, of which Firmicutes (64%), Bacteroides (28%), Proteus (8%), and Actinomycetes (3%) account for 98% of the flora (83). Dietary compounds indirectly affect immune responses by regulating the microbiota composition. For instance, insoluble dietary fiber extracted from barley leaves increased *Parasutterella* and *Alistipes* abundance to a certain extent and decreased *Akkermansia* abundance, as well as markedly relieved acute colitis symptoms and decreased levels of inflammatory factors such as IL-6, TNF-α, and IL-1β in colitis mice. In addition, short-term rice bran consumption reduces the Firmicutes: Bacteroidetes ratio in humans but may increase it in the long run (84). Therefore, there may be a time difference in the impact of dietary compounds in reducing CRC risk (85). Additionally, genetically modified mice with the same epigenome but two different gut microbes were fed four equal-calorie diets with the same dietary fiber composition. Integrated transcriptomic and metabolomic analyses showed that the metabolic results of the final amino acids and lipids in each group were different, indicating that gut microbiota structures also affected metabolism of dietary compounds (86).

### Metabolic regulation of intestinal flora on dietary compounds

The metabolism of dietary compounds by gut microbiota is mainly catabolic, which reduces the molecular weight of drugs, weakens polarity, and increases fat solubility and the efficacy. According to research on ginseng metabolism, microbiota can metabolize the ginsenosides Rb1 and RD into compound K, which has a potent anticancer effect (87). The oral bioavailability of food components can also be improved by gut flora. Curcumin can prevent tumor growth *in vivo* by increasing the chemical sensitivity of HCC cells to 5-FU *via* blocking the G2/M phase of the cell cycle, and reducing the activation of downstream protein kinases in the PI3K/AKT/mTOR signaling pathway (88). Glycyrrhizin is a flavonoid food component that can be metabolized by gut flora to yield three potential metabolites: pantothenic acid (M3), resorcinol (M4), and M5 to achieve antitumor activity (89, 90).

## Interactions between TCM and gut microbiota

TCM is mostly used in decoctions. The main components of TCM ingested by the human body from such decoctions include polysaccharides, peptides, flavonoids, alkaloids, polyphenols and anthocyanins. As specific components are more complex than those of dietary compounds, TCM has multi- component, level, and target effects. The herbal medicine WangShiBoChiWan (WSBCW) increases the number of *Bifidobacterium* and *Desulfovibrio*, restrains *Bacteroides fragillis* in the gut, upregulates intestinal junction proteins, increases long villi length, and reduces the levels of inflammatory factor (91). XiaoChaiHuTang (XCHT) partially reverses gut dysbiosis associated with CRC progression inhibition, and the mechanism may be related to the TLR4/MyD88/nuclear factor (NF)-κB downregulation of signaling pathways (92). Fecal microbiota transplantation from GeGen QinLian decoction (FMT-GQD) treatment inhibits nucleotide-binding and regulates important pathways, including the oligomerization domain (NOD), receptor-interacting-serine, threonine-protein kinase 2 (RIP2), and NF-κB signaling pathways, which could influence the expression of related downstream inflammatory factors and inhibit the activation and differentiation of CD4^+^ T cells to influence the immune system (93).

**Figure 3:**
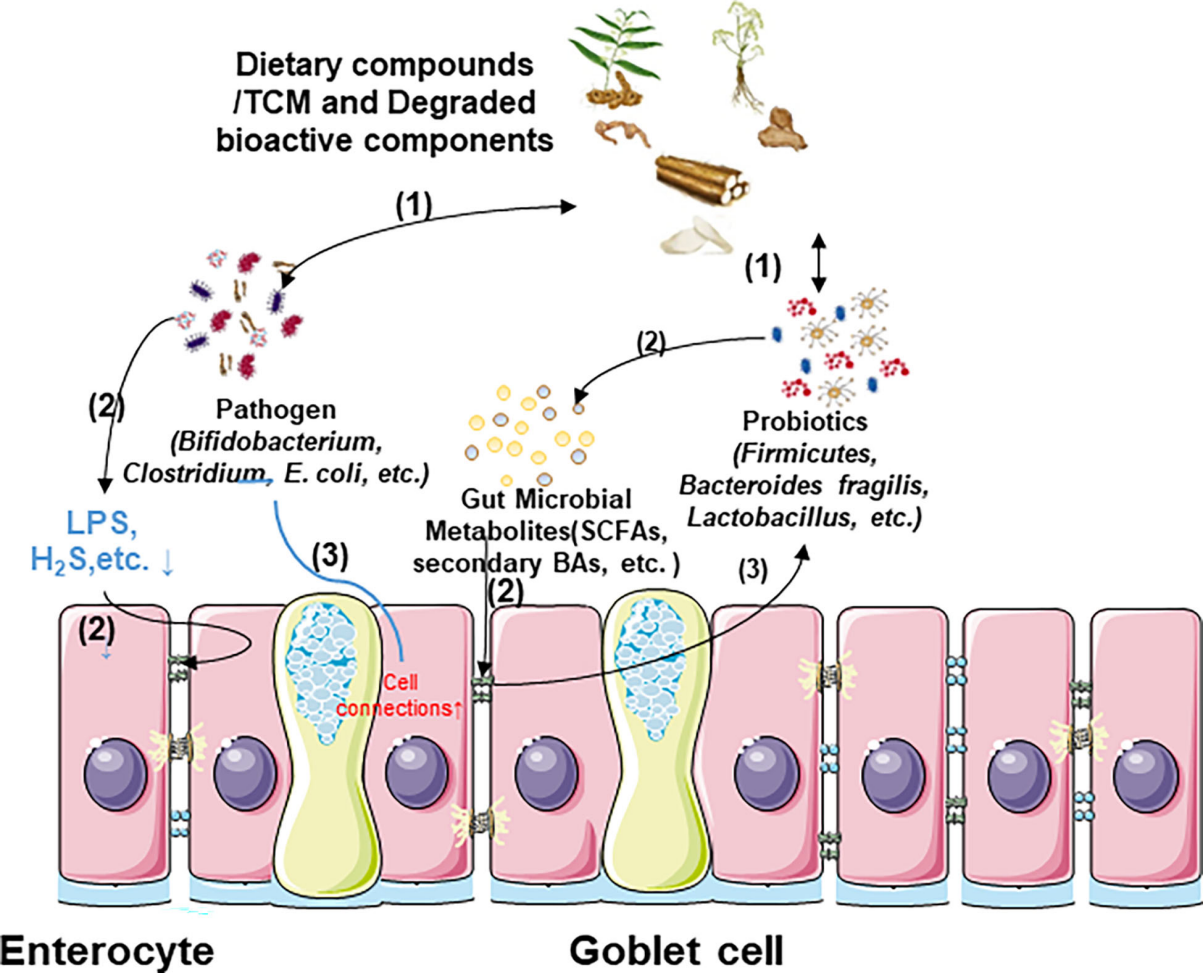
Relationship between dietary compounds/TCM and gut microbiota. (1) Dietary compounds/TCM and degraded bioactive components can modulate the microflora structure, and the different composition of gut flora among species also influences their curative effect. (2) Some gut microbial metabolites produced by probiotics restore intestinal epithelial cell barrier, and the reduction of harmful metabolites produced by pathogens also decreases intestinal barrier damage. (3) Cell barrier restoration is also conducive to gut flora stability.

Gut bacteria can produce a large number of enzyme systems, mainly including glucuronidase β-glucose enzyme, nitroreductase, and protease to degrade and release a variety of active ingredients that are convertible, directly altering the toxicity of TCM (94). By diacylation, esterification, and elimination of methyl hydroxyl, the intestinal microbiota can transform aconitine, the primary poisonous component of aconite, into monoester diterpene alkaloids that act as anti-inflammatories (95). However, the negative effects of gut flora that increase TCM toxicity are also significant. The hepatotoxic and carcinogenic effects of cycasin, an azo-glucoside found in the plants of the Cycadaceae family, may be caused by the microflora modulation *via* converting it into three different carcinogens, diazomethane cycasin, diazomethane cycasin, and cycasin (96). Consequently, comprehensive research into the dual effects of gut flora metabolism on TCM is necessary for digestive cancer immunotherapy. The relationships between dietary compounds/TCMs and intestinal microflora have been demonstrated in **Figure 3**.

## Dietary compounds/TCM regulate immunotherapy through interstinal microbiota intervention

Many active components of dietary compounds/TCM participate in regulating cancer immunotherapy, which may be linked to improved intestinal health and microbial metabolites. The mechanisms have been detailed in **Tables 1**, **2**.

**Table 1:** Dietary compounds improves immunotherapy by modulating intestinal microbiota.

Type	Dietary Compounds	Cancer Model	Bacterial population	Cancer Suppressive Effect	Reference
dietary fiber	Pectin	CRC	*Lachnoclostridium*(↑)*, Ruminococcaceae* (↑)*, Faecalibacterium* (↑)*, Subdoligranum* (↑)	tumor volume (↓), SCFA (↑), T cells infiltration (↑)	Zhang et al., 2021 (97)
	Inulin gel	CRC	*Akkermansia* (↑)*, Lactobacillus* (↑)*, Roseburia* (↑)	cytokines (↑), SCFA (↑), γ-IFN and CD8^+^T cells responses (↑)	Ni et al., 2020 (98)
	Albuca Bracteate Polysaccharides	CRC	*Alistipes* (↑)*, Roseburia* (↑)*, Ralstonia* (↓)*, Staphylococcus* (↓)	spleen index (↓), β-Catenin (↓), SCFA (↑)	Yuan et al., 2021 (99)
	Fucoidan	CRC	*Alloprevotella* (↑)*, Prevotella* (↓)	LPS (↓), β-Catenin (↓), SCFA (↑)	Li et al., 2022 (100)
	Cellulose	CRC	*Saccharimonas* (↑)*, Streptococcus* (↓)*, Eubacteriaceae* (↓)*, Clostridioides* (↓)	tumor volume (↓), colon length (↓), proinflammatory cytokines (↓)	Li et al., 2021 (101)
	Apple polysaccharide	CRC	*Bacteroides* (↑)*, Firmicutes* (↓)	tumor volume (↓), inflammation (↓), β-catenin (↓)	Li et al., 2021 (102)
flavonoids	Apigenin	CRC	*Actinobacteria* (↑)*, Bifidobacterium* (↑)*, Lactobacillus* (↑)*, Firmicutes* (↓)	tumor volume (↓), proinflammatory cytokines (↓), SCFA (↑)	Bian et al., 2020 (103)
	Corylin	CRC	*Bacteroidetes* (↑)*, Patescibacteria* (↑)*, Enterorhabdus* (↑)*, Firmicutes* (↓)*, Turicibacter* (↓)*, Romboutsia* (↓)	colon length (↑), serum C-reactive protein (↑), TLR4/p38/AP-1 signaling pathway (↓), M1/M2 macrophage polarization (↓)	Wang et al., 2022 (104)
	Bound polyphenol of the inner shell	CRC	*Bacteroides* (↑)*, Firmicutes* (↓)	tumor volume (↓), SCFAs (↑), indole (↑), β-Catenin (↓)	Yang et al., 2020 (105)
	Carnosic acid	CRC	*Bacteroides* (↑)*, Firmicutes* (↓)	proinflammatory cytokines (↓), SCFAs (↑),	Li et al., 2022 (106)
glycosides	Ginsenoside Rk3	HCC	*Bacteroidetes* (↑)*, Akkermansia* (↑)*, Lactobacillus, Lachnospiraceae* (↑)*, Firmicutes* (↓)*, Proteobacteria* (↓)	LPS/TLR4 signaling pathway (↓), number of hepatic nodules (↓), cytotoxic T cells (↑)	Qu et al., 2021 (107)
	Neohesperidin	CRC	*Firmicutes* (↑)*, Bacteroides* (↓)	tumor volume (↓), T cells infiltration (↑)	Gong et al., 2019 (108)
biological pigments	Bilberry anthocyanin	CRC	*Akkermansia* (↑)*, Bifidobacterium* (↑), *Clostridia* (↑)*, Lactobacillus* (↑)	tumor volume (↓), SCFA (↑), T cells infiltration (↑)	Wang et al., 2020 (109)
	recombinant phycoerythrin	H22 hepato celluar carcinoma	*Bacteroidetes* (↑), *Allpprevotella* (↑), *Alistipes* (↑), *Ruminococcus* (↑), *Firmicutes* (↓), *Prevotella* (↓), *Barnesiella* (↓)	tumor weight (↓), sphingolipid metabolism (↑), SCFA (↑)	Qi et al., 2019 (110)
	Safflower yellow	HCC	*Barnesiellaceae* (↓), *Erysipelotrichaceae* (↓), *Firmicutes* (↑), *Bacteroidetes* (↑)	number of hepatic nodules (↓), ALT, AST and ASP levels (↓), weight (↑)	Fu et al., 2021 (111)

Arrows ↑ or ↓ represent up-regulation or down-regulation.

**Table 2:** TCM improves immunotherapy by modulating intestinal microbiota.

TCM	Cancer Model	Bacterial population	Cancer Suppressive Effect	Reference
Gegen Qinlian decoction	CRC	*Erysipelotrichaceae* (↑), *Lactobacillus* (↑), *Parasutterella* (↑)	inflammation (↓), oxidative stress (↓), glycerophospholipid metabolism (↑), sphingolipid metabolism (↑)	Deng et al., 2021 (93)
WangShiBoChiWan	CRC	*Bifidobacterium* (↑), *Prevotellaceae* (↑), *Bacteroides* (↓), *Lachnospiraceae* (↓), *Firmicutes* (↓)	tumor volume (↓), spleen weight (↓), inflammation (↓), Wnt/β-catenin signaling pathway (↓)	Yin et al., 2021 (91)
Canmei formula	CRC	*Desulfovibrionaceae* (↓), *Rikenellaceae* (↑), *Alistipes* (↑), *Turicibacter* (↑), *Bacteroides* (↑), *Faecalibaculum* (↑)	tumor volume (↓), proinflammatory cytokines (↓), NF-κB signaling pathway (↓)	Zhang et al., 2019 (112)
Spore Powder	CRC	*Firmicutes* (↑), *Bacteroides* (↓)	inflammation (↓), cytotoxic T cells (↑)	Yang et al., 2022 (113)
Pai-Nong-San	CRC	*Firmicutes* (↑), *Bacteroidetes* (↓), *Proteobacteria* (↓), *Lactobacillus* (↓)	alleviation of hematochezia, DAI score (↓), colon length (↑)	Zhang et al., 2020 (114)
Wu Mei Wan	CRC	*Bacteroidetes* (↓), *Firmicutes* (↑), *Lachnospiraceae* (↑)	tumor volume (↓), tumor marker expression (↓), NF-κB/IL-6/STAT3 pathway (↓)	Jiang et al., 2020 (115)
Gui Shao Tea	Gastric Cancer	*Firmicutes* (↑), *Proteobacteria* (↑), *Bacteroides* (↓)	induce tumor cell apoptosis and cell differentiation, block cell cycles	Liu et al., 2022 (116)
Siwu-Yin	Esophageal Precancerous Lesions	*Turicibacter* (↑), *Bacillus* (↑)	spleen index (↓), regulate the synthesis and secretion of bile acids to affect macrophage polarization	Shi et al., 2022 (117)
Danggui Buxue Decoction	CRC	*Odoribacter* (↓), *Helicobacter* (↓), *Lactococcus* (↓), *Alloprevotella* (↓), *Ruminococcaceae* (↑), *Odoribacter* (↑)	tumor volume (↓), SCFA (↑)	Shi et al., 2021 (118)

Arrows ↑ or ↓ represent up-regulation or down-regulation.

### Dietary fiber

Pectin, a soluble fiber extracted from plant cell walls, can be used to block the proliferation cycle of tumor cells, thereby inhibiting CRC (97, 119). The gut flora of CRC subjects in tumor-bearing mice was dosed orally with pectin, which significantly improved the anti-PD-1 monoclonal antibody effect (82). Moreover, mice treated with gut flora from patients resistant to anti-PD-1 antibody showed a similar effect. Inulin is a plant-derived polysaccharide fiber present in Jerusalem artichoke and chicory tubers. It can be adsorbed into intestinal mucosa folds through the thickening effect, which is conducive to its interaction with gut flora. *Akkermansia*, *Lactobacillus*, and *Rosebacilli* are the main SCFA-producing bacteria, and their abundance increased significantly after inulin gel administration. Notably, high-dose inulin intake for 14 days (approximately 450 mg per day) slows tumor growth in mice before tumor modeling but does not work in synergy with anti-PD-1 (98). This may be related to a reduction in gut flora diversity caused by a single diet. Approximately 90% CRC tumors exhibit abnormal Wnt/β-catenin pathway activation (120). Albuca Bracteate Polysaccharides (ABP) treatment inhibits β-catenin expression. Furthermore, ABP and 5-FU (first-line drugs for CRC treatment) synergistically affected CRC tumor cell growth, migration, and invasion, and the antitumor effect of their combination was better than that of 5-FU or ABP alone. Fucoidan significantly increased *Lactobacillus* levels and decreased *Fusobacterium* levels in CRC mouse models, which alleviated macrophage and T cell infiltration, and reduced colonic inflammation (100). Moreover, fucoidan also inhibited the Wnt/β-catenin pathway (121). Dietary fibers with similar effects also contain cellulose and apple and jujube polysaccharides, which reduce the abundance of differential bacteria correlated with IL-6, IL-1β, and TNF-α concentrations to inhibit colon tumor formation (101, 102, 122).

### Flavonoids

Corylin is one kind of flavonoids isolated from the fruit and seed of *Psoralea corylifolia* (104). Corylin improved intestinal homeostasis to further reduce tumor cell-induced inflammation by inhibiting the TLR4/p38/AP-1 pathway, inflammatory factors, and the contact between bacteria and epithelial cells in CRC mice. Apigenin has been reported to prevent atrophic gastritis and subsequent gastric cancer caused by *Helicobacter pylori*. Apigenin also treats tumors by regulating gut flora associated with SCFA production, such as *Bifidobacterium* and *Lactobacillus* (103, 123). Bound polyphenol of the inner shell (BPIS) sharply restores *Lactobacillus* and *Bifidobacterium* abundance in the intestine of CRC mouse models, with an increase in various lymphoid subgroups such as CD4^+^, CD8^+^T cells, and NKT cells in the blood (105). BPIS also increases the number of microbiota products, including SCFAs and indole derivatives, which boost intestinal junction recovery and alleviate inflammation in tumor-bearing mice (124). The above result shows that BPIS can regulate oncogenic inflammation. The initial response to harmful stimuli is acute inflammation, with chronic inflammation potentially resulting from the persistence of inflammatory factors (125). Inflammatory cells and cytokines act as tumor promoters during chronic inflammation, affecting cell survival, proliferation, invasion, as well as angiogenesis. Moreover, the effect of inflammation on most cancers is double-edged, since cancer also affects inflammation. Inflammation has a close relationship with tumors, making inflammation an important target for anticancer treatment (126). Activating anti-cancer immunity cells can improve the cancer-killing ability of the immune system (127, 128). These results showed that BPIS may become a new cancer drug through microbial restoration and immune regulation. Other flavonoid compounds with anti-tumor effects include carnosic acid (106) and baicalin (129).

### Glycosides

An early study showed that Ginsenoside Rk3 exerts antitumor effects *in vitro*, without toxic effects on normal cells and lymphocytes (105). Later, it was found that a crucial factor in avoiding HCC is the LPS-TLR4 signaling pathway that Ginsenoside Rk3 inhibits by enhancing gut microbial imbalance (107). The mechanism of Rk3 against esophageal cancer is also related to this pathway. Neohesperidin (NHP), found in citrus fruits, upregulates *Firmicutes* and *Proteobacteria* and downregulates *Bacteroides* abundance. Furthermore, NHP treatment significantly increases IFN-γ expression and CD4^+^ and CD8^+^ T cell infiltration in mouse tumor cells, whereas the antibiotic cocktail (ABX) suppresses this effect (108). Consequently, NHP, a microecological regulator, induces antitumor immunity by improving immune checkpoint efficacy as a glycoside.

### Biological pigments

Anthocyanins are polyphenolic compounds widely present in plants. Bilberry anthocyanin extracts (BAE) assist in systemic nutritional status and immunity improvement by increasing the levels of *Lachnospiraceae johnsonii* in Firmicutes (109). Meanwhile, BAE induces T cell responses and increases SCFA production by upregulating Clostridia to ferment resistant starch and nonstarch polysaccharides. Simultaneously, the ratio of aerobes decreased remarkably, and the proportion of anaerobes increased with BAE, showing that oxygen content reduction may help BAE improve the microflora environment. Recombinant phycoerythrin (RPE) is a light-harvesting pigment that greatly reduces tumor weight, increases the incubation period of tumor cells, and inhibits cancer growth in H22-bearing mice. Moreover, RPE improved the probiotic level and sharply reduced the pathogen level compared to the cyclophosphamide group (110). Safflower yellow (SY), the main active ingredient from *Carthamus tinctorius*, modulates microbiota composition in BC mice, including increased *Bacteroides fragilis* and *Clostridium* counts, which could suppress pro-inflammatory factor activity and induce CD8^+^T cell and butyric acid production. This finding provides evidence that SY improves the immune microenvironment by affecting the immune cell components of the liver and modulating the abundance of inflammation-related gut microbiota (111).

Furthermore, combined treatment with multiple dietary compounds requires more attention. Combined GLP and GPS treatment significantly improved the intestinal barrier by blocking colonic polyp growth, transforming M1 to M2, effectively adjusting epithelial–mesenchymal transition markers and cutting carcinogenic signaling molecules in Apc^Min/+^ mice. Their combination also greatly promotes SCFA-producing bacteria and inhibits sulfate-reducing bacteria (130). However, some highly disconcerted effects were also observed. Soluble fermentable fiber inulin may lead to gut flora imbalance and icteric hepatic cellular cancer (131). Excessive inulin increases SCFA production and stimulates immune cells to produce inflammatory factors, including IL-1α, IL-1β, IL-6, and IL-10, which cause acute lamellar inflammation and laminitis. Therefore, the application of dietary compounds in immunotherapy requires more comprehensive investigations.

### TCM

GQD is a classical TCM formula, and its active compounds, including baicalin, Glaxo, and berberine, greatly reduce the inflammatory response and oxidative stress both *in vivo* and *in vitro*, which have been used in ulcerative colitis therapy (132, 133). Additionally, GQD and anti-PD-1 combination therapy downregulates PD-1 and increases IL-2, indicating that the combined treatment restores T-cell function to a certain extent by suppressing the checkpoint blockade. Combination therapy also increased Bacteroides groups, which reduces the pro-inflammatory activity of the mouse small intestine and exerts immune regulation by releasing extracellular bacterial DNA. Notably, combination therapy changes glycerophospholipid and sphingolipid metabolism, which could be used as biomarkers for monitoring patients with CRC (134).

Experimental results have indicated that sporoderm-broken *Ganoderma* reverses the tumor xenotransplantation-mediated microbiota structural shift (135) by increasing the immunoactivity-related genera, and by reducing microbiota, such as Bacteroides, which cause immunologic suppression and carcinogenic effects (136). The transformation of microbiota leads to changes in a series of key metabolites, including several amide acids necessary to form SCFAs. The Yiyi Fuzi Baijiang decoction (YFBD), composed of *Coix* seed and *Patrinia villosa*, is a TCM used to treat gastrointestinal disorders. *Coix* seeds and *Patrinia villosa* demonstrated antitumor hyperplasia effects in several carcinoma cell lines (137, 138). YFBD inhibited CRC cell proliferation and development in Apc^Min/+^ mice without significant weight changes or immune recovery. YFBD also changes Apc^Min/+^ mice intestinal bacteria, such as *Bacteroides fragilis* and *Trichospiroideae*, which regulate the Treg/Th17 ratio to control carcinogenesis (139).

Siwu-Yin inhibits esophageal precancerous lesion occurrence by increasing *Turicibacter* abundance, regulating bile acid synthesis and secretion metabolic pathways, and improving macrophage polarization (117). Accordingly, TCM has great potential in preventing digestion cancer progression in addition to its application in tumor immunotherapy. Furthermore, Danggui Buxue decoction (DBD) significantly improves bone marrow suppression-mediated anemia after CRC chemotherapy, while treating CRC by increasing butyric acid-producing bacterial abundance (118). This suggests a novel use of TCM for treating the postoperative side effects of digestive cancer.

## Conclusions

With the development of 16S rRNA high-throughput sequencing, TCM microencapsulation, and CAR-T technology, we have reached a new level of understanding regarding the modulation of gut flora by dietary compounds/TCM for digestive cancer immunotherapy. Dietary compounds/TCM are primarily metabolized in the intestine. Long-lasting effects on the gut microbiota from dietary compounds/TCM can strengthen the biological function *via* their conversion into bioactive metabolites. The studies mentioned above provide prospective methods to arrest tumor progression by enhancing the intestinal barrier, inhibiting pathogens, restraining inflammatory reactions, provoking tumor cell apoptosis and metabolism, and controlling SCFA secretion. However, research must overcome some obstacles.

Firstly, most research focused on the correlation analysis of dietary compounds/TCM on the structure and composition of gut flora, intestinal immune inflammatory reaction, intestinal barrier function and bacterial metabolites, but further multi-channel research has been neglected. In addition, the vast majority of experimental designs only stay in one stage, such as in precancerous lesions of digestive tract tumors, or in the tumor development or pre-late stages. We believe time series analysis is required to conduct a longitudinal survey on the tumor immune methods of dietary compounds/TCM modulating gut microbiota, which may provide more information. Notably, most digestive tumor models were conducted on only one strain of mice, resulting in a lack of comparisons and inductions between different strains of mice. Furthermore, the therapeutic effects of dietary compounds/TCM in different species (such as rabbits, monkeys, etc.) should also be investigated.

We recommend that future research should focus on the following aspects. Firstly, some dietary compounds/TCMs have shortcomings such as low bioavailability and bioactivity, toxic or side effects, and insufficient supply, which remain the main obstacles to clinical transformation. Secondly, exactly which specific species of gut microbiota, or even which specific enzymes in the microbiota can metabolize dietary compounds/TCM remains to be elucidated. Moreover, the specific components of dietary compounds/TCM that play an immunomodulatory role after being metabolized should also be investigated. It is necessary to identify more precise targets to reduce the increased toxicity of dietary compounds/TCM after metabolism. Thirdly, regarding some TCM with complex prescriptions, it may be difficult to standardize research materials, because factors such as different processing methods or different raw material origins can affect the quality. Fourthly, since the therapeutic response of dietary compounds/TCM is affected by different digestive cancer heterogeneity, more information is needed to select the proper treatment and mode of natural compound administration. Therefore, more studies are required focusing on the effect of one specific type of digestive tumor or a single component of dietary compounds/TCM on tumor immunotherapy. Fifthly, the modification of dietary supplements/TCM, in comparison to western drugs, is still poorly understood, which make it more difficult to achieve selective targeted drug delivery, leading to many uncertainties about the efficacy of this method. We suggest that interdisciplinary methods, such as nanomedical technology and precision medicine, are required to improve the safety of dietary compounds/TCMs and achieve better therapeutic effects. These efforts will pave the way for the use of dietary compounds/TCMs in clinical researches. In summary, we hope this review will help to understand the specific mechanisms by which dietary components/TCM improve immunotherapy based on microbiota and provide a theoretical basis for the development of new drugs for treating malignant digestive tumor growth, recurrence, and complications.

## Author contributions

XF, WG and YH contributed to conception and design of the study. ZL organized the database. XF and ZL wrote the first draft of the manuscript. All authors contributed to manuscript revision, read, and approved the submitted version.

## Glossary

**Table:**

ABP	Albuca Bracteate Polysaccharides
ABX	antibiotic cocktail
ADCC	antibody-dependent cellular cytotoxicity
AHR	aromatic hydrocarbon receptors
AOM/DSS	azoxymethane/dextran sulfate sodium
APS	Astragalus polysaccharides
BAE	Bilberry anthocyanin extracts
HCC	hepatocellular carcinoma
BFT	Bacteroides fragilis enterotoxin
BPIS	Bound polyphenol of the inner shell
NHP	Neohesperidin
CMF	Canmei formula
CRC	colorectal cancer
CTLA-4	cytotoxic T lymphocyte-associated antigen-4
DBD	Danggui Buxue decoction
ESG	sporoderm-broken Ganoderma
FMT-GQD	fecal microbiota transplantation from GeGen QinLian decoction
GLP	gypenoside
GPCR	G protein-coupled receptors
GPS	Ganoderma lucidum polysaccharides
HDAC	histone deacetylase
ICB	immune checkpoint blockade
IDO	indoleamine 2,3-dioxygenase
Ig	immunoglobulin
IL	interleukin
MAPK	mitogen-activated protein kinase
MDSC	Myeloid-derived suppressor cell
NF	nuclear factor
NK	natural killer
NOC	N-nitroso compound
HCC	hepatocellular carcinoma
MMPs	matrix metalloproteinases
NOD	nucleotide-binding and oligomerization domain
Nos2	type 2 NO synthase
PD-1	programmed cell death 1
PD-L1	programmed cell death ligand 1
PE38	Truncated *Pseudomonas* exotoxin
PI3K	phosphatidylinositol-3-kinases
RIP2	receptor-interacting-serine/threonine-protein kinase 2
RPE	recombinant phycoerythrin
SCFA	short-chain fatty acids
SY	Safflower yellow
T-ALL	Acute T lymphocyte leukemia
TCM	Traditional Chinese Medicine
TGF	transforming growth factor
TH	T-helper
TIL	tumor infiltrating lymphocytes
TLR	toll-like receptor
TNF	tumor necrosis factor
